# Future Perspectives of Therapeutic, Diagnostic and Prognostic Aptamers in Eye Pathological Angiogenesis

**DOI:** 10.3390/cells10061455

**Published:** 2021-06-10

**Authors:** Emilio Iturriaga-Goyon, Beatriz Buentello-Volante, Fátima Sofía Magaña-Guerrero, Yonathan Garfias

**Affiliations:** 1 MD/PhD (PECEM) Program, Facultad de Medicina, Universidad Nacional Autónoma de México, Mexico City 04510, Mexico; iturriemilio@gmail.com; 2 Cell and Tissue Biology, Research Unit, Institute of Ophthalmology, Conde de Valenciana, Chimalpopoca 14, Mexico City 06800, Mexico; bbuentello@institutodeoftalmologia.org (B.B.-V.); fatima.magana@institutodeoftalmologia.org (F.S.M.-G.); 3 Department of Biochemistry, Facultad de Medicina, Universidad Nacional Autónoma de México, Av. Universidad 3000, Mexico City 04510, Mexico

**Keywords:** molecular-targeted therapy aptamers, angiogenesis, nucleolin, pathological angiogenesis, SELEX, diabetic retinopathy

## Abstract

Aptamers are single-stranded DNA or RNA oligonucleotides that are currently used in clinical trials due to their selectivity and specificity to bind small molecules such as proteins, peptides, viral particles, vitamins, metal ions and even whole cells. Aptamers are highly specific to their targets, they are smaller than antibodies and fragment antibodies, they can be easily conjugated to multiple surfaces and ions and controllable post-production modifications can be performed. Aptamers have been therapeutically used for age-related macular degeneration, cancer, thrombosis and inflammatory diseases. The aim of this review is to highlight the therapeutic, diagnostic and prognostic possibilities associated with aptamers, focusing on eye pathological angiogenesis.

## 1. Introduction

In recent years, clinical studies have confirmed that targeted therapy possesses higher affinity, specificity and selectivity properties compared to classic antibody therapy. Molecular-targeted therapy has changed the course of human therapy since immunotherapy arose. Targeted therapy has lower toxic outcomes compared to conventional chemotherapy, also, it has a wide range of chemical conjugation possibilities to other molecules, improving drug serum half-life, tissue penetrating capabilities and inhibiting serum drug degradation [1,2,3]. This novel therapy acts due to different mechanisms of action, such as downregulation of cell cycle, induction of apoptosis, targeting angiogenesis pathways, enzyme inhibition, neutralization of diverse human growth factors and serves as a platform for drug delivery systems, among many others. Positive therapeutic outcomes have been reported with targeted therapy in pathological angiogenesis. Pathological angiogenesis is involved in many diseases, including cancer [4,5,6], metastasis [7], obesity [8,9], joint synovial neovascularization secondary to rheumatoid arthritis [10,11], enlargement of existing vessels in papillary dermis secondary to psoriasis [12], persistent hyperplastic vitreous syndrome due to genetic loss of angiopoietin-2 (Ang-2) [13], plaque angiogenesis in atherosclerosis [14], asthma [15], preeclampsia [16], COVID-19 [17,18] and eye diseases [19].

## 2. Mediators of Ocular Angiogenesis

Retinal ischemia and intraocular neovascularization are long-term consequences of pathological angiogenesis, which generate a destructive cascade involving neuronal depolarization, calcium influx and oxidative stress initiated by anaerobic metabolism, energy failure and an increased glutamatergic stimulation. There are different ocular pathologies that produce ischemic cascade, such as proliferative diabetic retinopathy (PDR) [20], age-related macular degeneration (AMD), central retinal vein occlusion [21] and retinopathy of prematurity (ROP) [22]. Clinical outcomes of intraocular neovascularization result in retinal detachment, vitreous hemorrhages, neovascular glaucoma and blindness [23,24,25,26,27,28,29,30].

During different disease states, cellular oxygen levels are often insufficient to deliver physiological requirements. Mammalian cells respond to hypoxia, modifying gene expression, increasing anaerobic energy, preventing stress cytotoxicity, regulating cell survival and augmenting local angiogenesis in order to compensate oxygen demands. In this context, when metabolically active cells grow beyond the oxygen diffusion threshold, hypoxia-inducible factor-1α (HIF-1 α) binds to the promoter region of diverse hypoxia-responsive family genes. This response produces an upregulation of angiogenic genes such as Ang-1/2 [31,32], erythropoietin (EPO) [33], basic fibroblast growth factor (bFGF), platelet-derived growth factor (PDGF) [34], transforming growth factor alpha (TGF-alpha) [35], interleukin-8 (IL-8) [36], connective tissue growth factor, insulin-like growth factor (IGF-1) [37], bone morphogenic protein (BMP) [38] and vascular endothelial growth factor (VEGF). Importantly, VEGF is one of the most common molecules of physiological and pathological angiogenesis [39,40,41]. VEGF was discovered by Dvorak et al., who observed that fibrin filaments were located outside tumorigenic blood vessels, and this finding supported the idea that tumor cells positively regulated vascular permeability [42]. Dvorak successfully isolated a protein that was named vascular permeability factor (VPF). A decade later, another group isolated an angiogenesis-inducing protein, which was called VEGF. Years later, researchers realized that VPF and VEGF were the same molecule, and interestingly, this molecule also promoted endothelial proliferation and hyperpermeability. The induction of a fenestrated endothelium phenotype promotes vascular hyperpermeability, and consequently protein leakage from blood vessels. Vascular hyperpermeability occurs preferentially in small blood vessels, where the microvascular architecture is characterized by a single layer of endothelial cells linked to each other by cell adhesion molecules. The cell adhesion molecules play a fundamental role in the endothelium, maintaining its barrier functions, such as the regulation of permeability. Plasma and protein extravasation function as a temporary scaffold in which migrating endothelial cells are able to proliferate and form new blood vessels from preexisting ones [43]. It has been shown that the molecular mechanism by which VEGF induces vascular hyperpermeability is through the activation of protein kinase C isoform β (PKC-β). This specific kinase induces phosphorylation and reorganization of the tight junction (TJ) complex, that forms a physical barrier preventing protein leakages from paracellular space and maintaining retinal homeostasis. PKC-β phosphorylates occludin (an important gap-junction protein) in multiple sites, including Ser490. The phosphorylation at Ser490 allows subsequent occludin ubiquitination and endocytosis, which disrupt the original architecture of the TJ, thereby generating endothelial hyperpermeability [44]. A clinical trial evaluated the PKC-specific inhibitor ruboxistaurin, which has demonstrated positive clinical outcomes in diabetic retinopathy (DR) and in macular edema [45,46].

## 3. Blood–Retinal Barrier (BRB) Dysfunction

The human retina has the highest oxygen consumption rate compared to all types of human tissues. Numerous types of retinal cells generate high metabolic rate, thereby the blood–retina barrier (BRB) is vital to regulate blood supply and oxygen demand. The BRB is composed of an inner and an outer barrier to warrant retinal homeostasis. The outer BRB is composed by the Bruch membrane and the retinal pigment epithelial (RPE), the latter of which provides nutritional requirements for photoreceptors, and RPE also has phagocytic activities and clean waste products of the outer segments of the photoreceptors [47]. The RPE is constituted by a single layer of pigmented cells situated underneath the neural retina and on top of the choroids. The RPE separates the neural retina from the fenestrated endothelium of the choriocapillaris. Nutrients, ions and water transportation, light absorption and protection against photo-oxidation [48] and visual cycle restoration by isomerization of all-*trans*-retinal into 11-*cis*-retinal [49], are some of the RPE functions. The RPE functions like a tissue resident phagocyte that maintains retinal homeostasis. A single RPE cell is in contact with approximately 30 photoreceptors and is responsible for the phagocytosis and removal of the distal portions of the photoreceptor outer segments (POS) [50,51]. The removal of the aged portions of the POS and of their metabolites is essential for visual cycle, retinal homeostasis, subretinal space composition and retinal cell survival. The neovascular form of AMD is characterized by the invasion of choroidal capillaries through subretinal space, thus affecting RPE homeostasis. This retinal disease promotes extravasation of serum components to the apical surface of the epithelium [52]. Dysfunctional TJ that are localized in the RPE facilitate serum leakiness and the spreading of the disease through the paracellular space. Retinal fluorescein angiography is a standardized method to evaluate retinal vascular leakage in clinical practice. Fluorescein leakage suggests outer BRB breakdown. On the other hand, the inner BRB resembles the blood–brain barrier. It is situated in the inner retinal microvasculature and is composed by endothelial cells [53]. The TJ in the endothelial cells generate a highly selective barrier that limits molecule diffusion and maintains inner retinal homeostasis [54]. The early stage of DR is characterized by inner BRB dysfunction, which dysregulates retinal homeostasis [55]. The inner BRB breakdown augments leukocyte extravasation and proangiogenic molecules’ recruitment, generating local inflammation. Numerous angiogenic molecules are produced by leukocytes and retinal homing cells, nevertheless, and as aforementioned, one of the most studied molecules is the VEGF. Importantly, it has been shown that VEGF levels in both vitreous and plasma are directly correlated with the progression of PDR after vitrectomy [56,57].

VEGF is produced by different types of cells, including platelets, macrophages, RPE, astrocytes, Müller cells, ganglion cells, endothelial cells, keratinocytes, tumor cells and hypertrophic chondrocytes, among many others [58,59]. Angiogenesis inducers promote vessel sprouting through inhibition of endothelial cell apoptosis, augmentation of matrix protease production and increasing endothelial cell motility [60]. 

It is hypothesized that retinal homeostasis is achieved by an equilibrium between pro- and anti-angiogenic molecules. The mechanisms of action of antiangiogenics are inhibiting the activity of proteases generated by angiogenic inducers, interfering with signal transduction produced by angiogenic inducers, and inhibiting endothelial cell proliferation, migration and tube formation [61]. One of the most important antiangiogenic factors is the pigment epithelium-derived factor (PEDF). PEDF was initially identified from cultured EPR, and currently, it is known that PEDF acts as a cell survival molecule [62]. Previous studies have confirmed that a decrease in the retinal levels of PEDF is associated with the formation and progression of pathological angiogenesis in the retina. It has been described that intravitreal administration of PEDF reduces retinal neovascularization and vascular hyperpermeability. Zhang et al. reported that PEDF intravitreal injection augmented occludin retinal levels compared to control eyes. Besides PEDF, there are other antiangiogenic factors in the retina that play an important role in maintaining the angiogenic homeostasis, such as the transforming growth factor beta (TFG-beta), endostatin [63], thrombospondin [64], somatostatin [65] and vasoinhibins [66]. Vasoinhibins maintain the quiescent state of retinal blood vessels and protect retinal cells against a dysregulated angiogenic environment in DR and ROP [67]. Vasoinhibins are antiangiogenic peptides synthetized from prolactin, growth hormone and placental lactogen in physiological conditions. Different mechanisms of action attributable to vasoinhibins have been described, such as preventing the activation of the MAPK pathway which induces cell-cycle arrest, inhibiting endothelial cell migration by increasing type-1 plasminogen activator inhibitor (PAI-1), and consequently reducing urokinase activity, and enabling endothelial cell apoptosis by promoting NFκB-mediated caspase-8 and 9 activation, among other mechanisms of action [68,69].

## 4. Physiological Angiogenesis and Pathological Angiogenesis 

Angiogenesis is the formation of new sprouting vessels from preexisting ones; in contrast, vasculogenesis arises from de novo production of endothelial cells, which primarily occurs in the embryo. Vasculogenesis is performed by endothelial precursor cells called angioblasts, these cells migrate and differentiate into new blood vessels. It has been shown that the formation of new blood vessels in adults occurs predominantly by angiogenesis, but vasculogenesis is also involved due to the migration of bone marrow-derived endothelial stem cells [70,71]. 

In order to generate new blood vessels from preexisting ones, firstly, quiescent endothelial cells sense an angiogenic signal. Pericytes detach from the endothelial basement membrane by secreting metalloproteinases. Endothelial cells loosen their junctions, and the nascent vessels dilate due to the pericyte loss. Then, a positive gradient of proangiogenic molecules stimulates the permeability of the endothelial cell layer, causing extravasation of plasma proteins that lay down and function as a provisional extracellular matrix (ECM) scaffold for the new sprouting vessels. Endothelial cells migrate onto the ECM scaffold, and proteases are secreted to release angiogenic factors that are bonded to heparin molecules situated in the ECM that represent a vital reservoir [72]. Angiogenesis requires hierarchical organization, this is achieved by specialized endothelial cells that lead the vascular growth, while most endothelial cells remain quiescent. This stretch organization is mediated by tip cells, and these cells are specialized endothelial cells located at the tip of growing vessels. Tip cells are motile cells that dynamically extend long filopodial extension to sense positive or negative signals for guidance [73]. Tip cells are not involved in the lumen vessel formation, instead, they guide the angiogenesis process and recruit perivascular cells to stabilize newly formed vessels [74]. On the other hand, lumen formation and vessel elongation are generated by stalk cells. Stalk cells proliferate and bridge the gap between the tip cells and the newer vasculature. The development of new blood vessels is crucial for embryonic growth and throughout life for physiological repair processes such as wound healing and post-ischemic tissue restoration, and also, during the menstrual cycle, angiogenesis occurs regularly in the corpus luteum and in the basal layer of the endometrium [75,76].

Under physiological circumstances, angiogenesis is a highly organized process. Excessive number of tip cells with short filopodia extensions that grow in all directions without any specific organization are characteristics of a disorganized and abnormal angiogenesis process widely described in PDR [77]. Tip cell molecular markers are VEGF receptor-2 (VEGFR2), Delta-Like protein-4 (DLL4), VEGFR3, PDGFB, endothelial cell-specific molecule 1 (ESM1), angiopoietin-2 (Ang2), CD34 [78,79] and low expression of VEGFR1 [80]; in contrast, stalk cell markers are VEGFR1, Jagged1 (JAG1) and Ang2 receptor [81]. Under VEGF stimulation, DLL4 expression is upregulated in the tip cells. DLL4 activates Notch signaling in the stalk cells, thus suppressing the tip cell phenotype. Notch signaling reduces VEGFR2 expression and increases VEGFR1 expression. 

The formation of a functional vascular network requires a tissue remodeling process, where tube-like structures are formed and organized by endothelial cells. Mural cells, such as pericytes in the microvasculature and smooth muscle cells in larger vessels, are recruited to the abluminal surface of the endothelium to provide stability and vessel maturation. Thus, vessels uncovered by pericytes regress. The vessel maturation process is mediated by angiopoietin-1 that binds to endothelial Tie-2 receptors [82]. However, tissue remodeling events can drive into physiological or pathological remodeling. Pathological tissue remodeling is an abnormal process occurring in different types of diseases or post-injury processes [83]. 

Pathological angiogenesis is characterized by abnormal endothelial cell division and upregulation of pleiotropic and/or synergic molecules related to hypoxia, inflammation and angiogenesis [84]. Cumulative evidence suggests that the upregulation of inflammatory factors such as tumor necrosis factor-α (TNF-α), intercellular adhesion molecule-1 (ICAM-1) and monocyte chemoattractant factor-1 (MCP-1 or CCL2), and subsequent leukocyte diapedesis, contribute to the BRB breakdown in diabetes. Numerous biomarkers have been proposed to understand the pathogenesis of abnormal angiogenesis; hence, they are being used as diagnostic, therapeutic and prognostic tools. There are molecules that are not or are minimally related with embryonic vascular development, but surprisingly are determinants of pathological angiogenesis, for example, cyclooxygenase-2 (Cox-2) [85], PLGF [86], αvβ3 [87], metalloproteinases [88], PAI-1 [89], nitric oxide (NO) [90], thrombospondin-2 [91,92], PECAM-1 or CD31 [93] have been related to pathological angiogenesis. 

Different types of cells modulate the angiogenic process, where vascular pericytes are periendothelial cells that support and wrap endothelial cells. Pericytes regulate blood flow by modulating capillary diameter due to contractibility mechanisms, vessel permeability, endothelial cell proliferation and leukocyte recruitment [94]. During capillary sprouting, pericytes migrate into the vessel wall due to paracrine communication by platelet-derived growth factor-B and its receptor (PDGF-B/PDGFRB) signaling. Importantly, in pathological angiogenesis, pericyte apoptosis produces vascular hyperpermeability. The first detectable sign of DR by fundus images is the presence of microaneurysms, caused by weakness of the capillary wall resulting in endothelial dilation and pericytes apoptosis. Microaneurysms are identified clinically by ophthalmoscopy as deep-red dots located in the retina [94,95]. Additionally, retinal pericytes regulate the expression of several genes (Ang2, VEGFR2 and Forkhead Box Protein O1 (FOXO-1)) to protect retinal vessels from injuries and stress, and a higher density of pericytes provides the micro-vessels with more resistance to damage. Additionally, pericyte–endothelial interactions promote the production and deposition of ECM components, including fibronectin, collagen and laminin [96]. Other types of cells involved in angiogenesis are fibroblasts, and these cells secrete soluble molecules that support endothelium sprouting and are necessary for vessel lumen formation [97,98].

## 5. Ocular Immunotherapy in Pathological Angiogenesis

Novel potential therapies have been available since molecular biotechnology revolutionized the conduct of human therapeutics. In a few decades, biotechnology has surprisingly evolved. The production of therapeutic monoclonal antibodies (mAbs) was developed due to the important discoveries by Köhler and Milstein in 1975 [99]. This technique consists in repetitively immunizing mice with a specific antigen and isolating the splenic plasma cells that recognize the antigen. Antigen-specific splenocytes are then fused with myeloma cells using electrofusion or polyethylene glycol (PEG) to immortalize the antigen-specific plasma cells. Hybridomas that produce specific antibodies are isolated and cloned. To reduce xeno-immunogenic responses, mouse sequences are replaced by human constant regions (humanized mAbs) [100]. However, humanization of mouse mAbs reduces antibody affinity. In contrast, phage display technology partially substituted hybridoma technology due to the possibility of obtaining higher affinity maturation, consequently improving therapeutic clinical outcomes [101]. 

mAbs have been used as therapeutic agents in many diseases. Bevacizumab is a recombinant humanized mAb with an immunoglobulin G1 conformation that is 93% human and 7% rodent. It binds with high affinity and specificity to VEGF. Bevacizumab potently neutralizes VEGF by interfering with VEGF-VEGFR1 and VEGFR2 signaling pathways. It was the first Federal Drug Administration (FDA)-approved anti-VEGF mAb for therapeutic purposes in metastatic colorectal cancer [102]. It has been used for non-small cell lung cancer, renal cell carcinoma, breast cancer and gastric cancer. Studies have determined that the efficacy of bevacizumab was related to the baseline expression of VEGF-A and neuropilin-1 in gastric carcinoma [103].

Bevacizumab was originally synthetized from a specific murine mAb using hybridoma technology. Evidence indicated that VEGF-A is an important mediator of vascular leakage and pathological angiogenesis, and several isoforms of VEGF-A are generated by alternative mRNA splicing. Ranibizumab, an antigen-binding fragment (Fab) that specially blocks VEGF-A, was obtained using phage display, improving VEGF-A binding due to the affinity maturation of the antibody. The molecular weight of ranibizumab is 48.39 kDa, compared to 149 kDa of bevacizumab [104,105,106]. Aflibercept is a human recombinant fusion protein composed of the second binding domain of VEGFR1 and the third binding domain of VEGFR2, with a total molecular weight of 115 kDa. These immunoglobulin-type fragments are fused to the Fc region of a human IgG1. This fusion protein is a “trap” molecule that catches, holds and blocks different cytokines. It binds to all VEGF-A isoforms, VEGF-B and PLGF [107]. The rates of endophthalmitis following intravitreal injections of aflibercept, bevacizumab and ranibizumab are 0.100%, 0.056% and 0.047%, respectively [108].

Preclinical studies showed that trastuzumab, a full-length IgG antibody, was not capable of crossing the retinal limiting inner membrane of the retina, thus, smaller molecules were proposed for treating PDR and AMD [109,110].

Brolucizumab is a humanized single-chain Fab that blocks all isoforms of VEGF-A. The molecular weight of brolucizumab is 26 kDa, a smaller molecule compared to bevacizumab, ranibizumab and aflibercept. In a randomized clinical trial in patients with AMD, brolucizumab presented lower intraretinal and subretinal fluid compared to the patients treated with aflibercept. The patients that were treated in the aflibercept arm received twice as many unscheduled injections and presented greater tachyphylaxis phenomena compared to the patients in the brolucizumab arm. These findings suggest a more durable blockage effect with brolucizumab than the already approved aflibercept [111,112,113,114]. It is important to mention that anti-VEGF resistance conduces to refractory or recurrent neovascular AMD, and hence other molecular-targeted therapies are being tested [115]. Moreover, systemic side effects such as thromboembolic events, hypertension, myocardial infarction, gastrointestinal perforation and cerebrovascular accident have been reported after long-term intravitreal anti-VEGF therapies, probably due to the BRB damage [104,116,117]. Anti-VEGF therapy produces local side effects that have been attributed to activation of fibrocytes, which promotes the formation of a fibrovascular membrane in the retina, producing retinal detachment and blindness [118].

## 6. Aptamers: Novel Oligonucleotide Therapy

Aptamers are single-stranded DNA (ssDNA) or RNA (ssRNA) molecules with variable length from 20 to 100 nucleotides. They are formed by complex and unique secondary and tertiary structures which confer enough recognition area and high range possibilities to interact with specific targets [119,120,121].

It has been widely studied that oligonucleotides recognize nucleic acid by base pairing, but surprisingly, aptamers create non-covalent bonds with multiple types of targets such as proteins, peptides, viral particles, vitamins, metal ions and even whole cells. Crystallization and structural determination of different aptamers have shown that hydrogen bonds, electrostatic interactions, hydrophobic and van der Waals forces confer high-affinity properties (ranges from μM to picoM Kd) [122,123,124]. Other types of oligonucleotide therapy are those containing immunostimulatory effects due to their CpG motifs. These CpG motifs enhance immune response by acting as agonists of Toll-like receptor 9, and therefore they could be used as vaccine adjuvants [125]. 

Aptamers are chemically synthesized oligonucleotides that are usually obtained from diverse nucleic acid libraries and selected by a method known as systematic evolution of ligands by exponential enrichment technology (SELEX). This method uses a random library of 10^13^–10^16^ ssRNA or ssDNA exposed to the target to be bonded. The oligonucleotides that are not attached to the target are discarded, while the target-bonded aptamers are then amplified using RT-PCR or PCR. This selection process is repeated 6–15 times to improve their affinity and specificity binding capabilities [126,127]. 

Moreover, aptamer customization has several advantages over mAbs. These advantages include reproducible synthesis between batches, easy and controllable post-production modifications [128], long-term stability in solution, non-toxic, non-immunogenic, room temperature stability, low costs of production, unlimited targets and the capability to be cell-internalized [129,130]. Aptamers can be conjugated with micelle particles to facilitate the entrance into the cells, which is performed by binding a lipid tail of phosphoramidite in a polyethylene glycol located in the aptamer end [131]. 

Standard IgG mAbs have an average mass weight of 150–170 kDa; in contrast, nucleotide aptamers are 10-fold smaller than mAbs, with 5–15 kDa. Proteins are molecules susceptible to lose their three-dimensional (3D) conformation when denatured at high temperatures, while nucleotide aptamers are thermally stable and maintain their 3D conformation, even with repeated denaturation/renaturation cycles. Aptamer applications in medicine are diverse, and they are currently being used for flow cytometry staining purposes, can activate signaling pathways through cell surface receptor ligation, serve as drug delivery systems, block protein–protein interactions and inhibit enzyme reactions, among others [132]. Drug delivery systems have a myriad of chemical conjugation potentials to aptamers. They function as nano-sized carriers using micelles, microspheres, liposomes, polymeric nanoparticles and nanomaterial-based enzymes (nanozymes). Microspheres made from poly-*D*, *L*-lactide (PDLLA) and polylactide-co-glycolide (PLGA) have been safely used as intravitreal drug delivery systems with adequate vitreous humor biodistribution [133]. Aptamer conjugation with nanomaterials such as nanozymes have emerged in recent years for developing biosensors to detect food contaminants, pesticides, pathogens and metal ions [134]. Nanozymes have attractive properties due to their high stability, low cost of production and longer-term storage. These properties confer potential applications in countries with unfavorable meteorological conditions and warmer temperatures. Nanozymes’ conjugation using affinity molecules such as aptamers and antibodies is a critical step for their production. Most usual types of conjugation are made by covalent or biotion/avidin linkages. [135]. 

Molecule customization has increased aptamers’ biodistribution and their renal clearance. Previous studies suggested that 3′-inverted thymidine modification in aptamer molecules increased the stability and resistance to 3′-exonucleases in human serum. The biotin conjugation to 3′ carbon atom resists the catalytic activity of 3′-exonucleases and slows down the clearance rate of aptamers from the blood circulation. Chemical modifications such as 2′-O-methyl (2′-OMe) increase aptamer serum half-life with in vivo stable conformations and reduce enzyme degradation [136,137,138,139,140]. 

In 2004, the FDA approved the first therapeutic RNA aptamer pegaptanib for the treatment of wet AMD that is characterized by pathological choroidal neovascularization. Pegaptanib stabilized vision and reduced the risk of severe visual loss in almost all patients with AMD, because it binds and blocks the VEGF-A_165_ isoform with high affinity and specificity. Pegaptanib intravitreally injected has a half-life of 10 ± 4 days, while ranibizumab has a half-life of 7 days, thereby pegaptanib could reduce the frequency of intravitreal administrations [141]. Studies suggested that bevacizumab can delay corneal wound healing, causing stromal thinning and affecting corneal homeostasis. Fortunately, new molecules have been found to reduce corneal neovascularization, such as a nucleolin-binding aptamer called AS1411 [142]. 

Molecular-targeted therapy is an emerging medical tool that has promising results in cancer and pathological angiogenesis. Encouraging results have been obtained by blocking immune checkpoints such as programmed death-ligand 1 and its receptor (PD-L1/PD-1) interactions [143]. This therapy has been used because PD-L1 plays a major role in suppressing the adaptive immune response. PD-1 is its receptor expressed on activated T cells, and the interaction with its ligand PD-L1 inhibits T cell response. PD-L1 is expressed on numerous cells, including epithelial cells, endothelial cells and immune system cells [144]. Importantly, PD-L1 is commonly upregulated on the surface of tumor cells, and this confers a tumor evasion mechanism to avoid apoptosis from lymphocytes. Thereby, poor clinical outcomes and low overall survival have been associated with the PD-1/PD-L1 pathway [145,146]. Targeted therapy that blocks the PD-1/PD-L1 immune checkpoint can enhance antitumor immunity by restoring anti-tumor cytotoxicity of activated T cells, producing a lasting clinical response and prolonging patient survival. Clinical studies indicate that therapies targeting PD-1 or PD-L1 can achieve promising results in numerous tumor types, including melanoma, prostate cancer and non-small cell lung cancer. The FDA has approved five monoclonal antibodies targeting this immune checkpoint, including atezolizumab, nivolumab, durvalumab, avelumab and pembrolizumab. Larger molecules such as antibodies led to lower tumor penetration, and therefore monoclonal antibodies could be substituted by aptamers due to their advantages. MP7 is a DNA aptamer that produces a specific antitumor response due to the inhibition of PD-1/PD-L1, diminishing tumor size in a colon carcinoma model [143,147]. Novel DNA nanostructures, named Holliday Junction (HJ), serve as carriers for drug delivery, nucleic acids and enzymes. This complex has a cross-type shape and is composed by four single-stranded DNA chains. This unique shape prevents HJ leaking out via renal clearance, augmenting its biodistribution. Recently, researchers have conjugated an aptamer with HJ (Apt-HJ) that blocks the PD-1/PD-L1 pathway. Interestingly, Apt-HJ had stronger affinity to colon cancer cells compared to the monovalent PD-L1 aptamer [144]. 

## 7. Nucleolin-Binding Aptamer, AS1411

Aptamers that contain G-rich sequences adopt a quadruplex conformation due to internal and/or inter-strand fold by hydrogen bonds. AS1411 is a guanine-rich aptamer that has been successfully tested in clinical trials with antineoplastic activity. AS1411 targets cell-surface nucleolin (NCL), a nucleolar multifunctional protein involved in organization nucleolar chromatin, packaging of pre-RNA and ribosome assembly, and its expression has been associated with proliferating cells. NCL localization changes in angiogenic vessels, and interestingly, surface and cytoplasmic NCL are differentiated from its nuclear counterpart by a slight change in their isoelectric point, which could be by N- and O-glycosylations and/or other post-translational modifications such as phosphorylation. In this context, our laboratory documented for the first time that NCL is able to translocate into the cell surface after angiogenic stimulus in an in vivo corneal neovascularization model [148]. Cell membrane NCL serves as a receptor for a diverse type of molecules, such as growth factors, laminin [149], P-Selectin [150], midkine [151], kallistatin [152], endostatin [153], pleiotrophin [154] and microorganisms such as HIV virus [155], *Helicobacter pylori* and *Escherichia coli* [156,157]. Surface NCL has been implicated in cell division, cell migration and adhesion, and participates in angiogenesis and tumor metastasis [142,158,159,160,161,162,163,164]. NCL has three structural domains: the N-terminal domain, the central domain and the C-terminal domain. The N-terminal domain has several long stretches of acidic residues involved in rRNA transcription. The central globular domain interacts with RNA-type molecules in four different sites, known as RNA-binding domains (RBD). The C-terminal domain contains nine folds of the tripeptide sequence arginine–glycine–glycine [165]. NCL positively or negatively modulates the turnover and transcription of diverse mRNA. NCL located in the cytoplasm binds to the 3′-untranslated region of the matrix-metalloproteinase-9 (MM-9) mRNA, and this interaction increases the production of the proteolytic enzyme (MM-9) that cleaves ECM components and promotes angiogenesis and tumor metastasis [166,167]. These regulations are driven by binding either mRNA 5′ UTR or 3′ UTR, producing negative translation or positive translation, respectively [168]. It has been shown that NCL can be phosphorylated by cyclin-dependent kinase-1 (CDK1), and this phosphorylation promotes NCL cytoplasmic localization, while non-phosphorylated NCL resides in the nucleolus. Another important protein is the non-muscle myosin heavy chain-9 (MyH9), that binds to NCL, functioning as a physical linker between NCL and the cytoskeleton, and this NCL–MyH9 association has been implicated in angiogenesis [158]. In our laboratory, we have described that AS1411 also inhibits cell migration of recombinant human (rh) VEGF-stimulated human limbal stromal cells (HLSC), and we have shown by transmission electron microscopy (TEM) that NCL was localized at the surface microvilli of rhVEGF-stimulated HLSC; moreover, we have proposed a possible mechanistic pathway in which the NCL–AS1411 interaction causes a reduction of the proangiogenic miR-21 and -221 [142]. Thus, we hypothesized that AS1411 could be beneficial as a treatment in eye pathological angiogenesis. 

Interestingly, human clinical studies in phase I reported good overall tolerability with no toxic effects [119]. Darche et al. reported that NCL expression was increased in endothelial cells of angiogenic retinal blood vessels compared to quiescent retinal blood vessels in mice. NCL localization was distributed on the nucleus of mature blood vessels, and surprisingly, extranuclear localization was found at the angiogenic front, specifically at the tip cell filopodia [159]. Surface NCL confers a tumor-selective affinity over AS1411, which preferentially targets the external site domain of NCL in cancer cells. The mechanism of the cytotoxicity of AS1411 is still being researched, but there have been many NCL-dependent and independent biological effects described. Methuosis is a nonapoptotic type of cell death characterized by cell vacuolization. Recently, methuosis has been linked with AS1411 aptamer therapy, due to the hyperstimulation of macropinocytosis and altered vesicle trafficking, producing cell death. AS1411 folds into diverse polymorphic G-quadruplex structures, which confers stabilization over pH fluctuations and serum nucleases, and consequently, increases cellular uptake efficacy. AS1411 have been covalently/non-covalently conjugated to a variety of nanoparticles. Shieh et al. created an aptamer-based anti-tumor therapy as a drug delivery system using photodynamic therapy to improve drug uptake in MCF7 breast cancer cells [168,169]. This was made by binding AS1411 to porphyrin TMPyP4 to increase drug uptake using photodynamic therapy. Recently, AS1411 has been studied as a supramolecular carrier for the delivery of an acridine-based G-quadruplex ligand named C_8_. Using flow cytometry, it was found that non-malignant cells presented lower complex internalization, which produced lower cytotoxicity towards non-malignant cells. This mechanism could be explained because nonmalignant cells lack a surface membrane NCL, and therefore the supramolecular carrier is being constantly eliminated by efflux or exocytosis, and the ligands cannot exert their cytotoxic effect [170]. Another type of drug delivery system using the AS1411 aptamer was described by Li et al., who used AS1411 as a molecular drug carrier to deliver siRNA in malignant melanoma treatment. This was achieved by binding a cationic liposome carrying a siRNA that silenced the *Braf* gen (SiBraf) to AS1411. As expected, the researchers found that SiBraf complex was able to downregulate the expression of human BRAF mRNA, therefore, the number of tumor cells was significantly reduced compared to controls [171]. SiRNA has been used for gene silencing, however the biggest challenge of gene therapy is the efficient delivery of exogenous genes or gene-modifying agents into the cells, thus molecular carriers are needed. Non-viral vectors with biodegradable materials can avoid immunogenicity compared to viral vectors. Liposomes are the most successful drug delivery system, because they can be conjugated to diverse types of ligands that specifically bind to molecules overexpressed in cancer and endothelial cells. Nevertheless, non-aptamer molecules have been tested for NCL inhibition, such as the pseudopeptide N6L, which decreased endothelial cell migration and tubulogenesis in different retinal disease models [159]. Talreja et al. proposed a platform for the delivery of diverse types of molecules, such as functional proteins into RPE, photoreceptors and ganglion cells using the nucleolin binding aptamer AS1411, to achieve therapeutic efficacy in an animal model of retinal disease [172]. 

**Figure 1:**
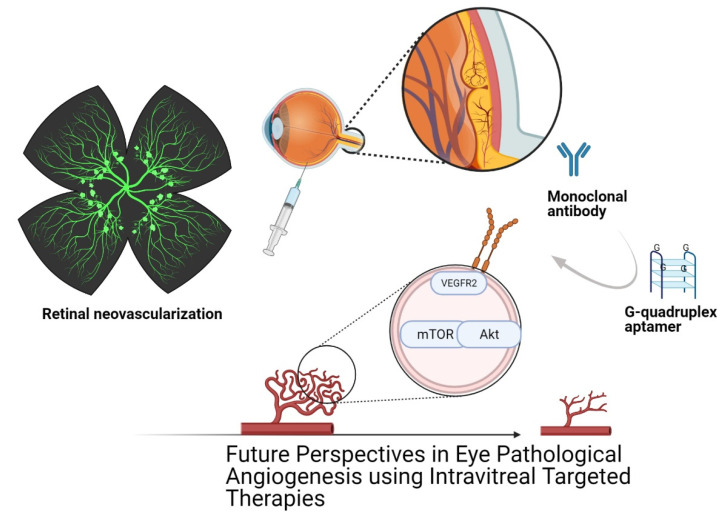
Future perspectives in eye pathological angiogenesis using intravitreal targeted therapy. Schematic retinal flat mount showing neovascularization on the superficial vascular plexus. Intravitreal eye injection is the therapy currently used as a treatment for proliferative diabetic retinopathy, retinopathy of prematurity, age-related macular degeneration and retinal vein occlusion. The treatments currently used focus on blocking the VEGF signaling pathway, and although it has beneficial results in patients, complications occur while inhibiting normal functions. Thus, researchers are looking for specific molecules that are not constitutively expressed that unbalance ocular homeostasis. G-quadruplex aptamers have many advantages compared to immunological therapy, such as lower molecular weight, controllable post-production modifications, non-toxic and non-immunogenic, with lower costs of production. Created with BioRender.com.

In recent years, intravitreal injection therapy against pathological angiogenesis consists in administrating drugs (steroids) or antibodies to reduce retinal edema and abnormal blood vessels; however, aptamers could be more efficacious to treat retinal angiogenesis due to their small size, high penetration in retinal tissue and due to the possibility of being administrated by intravenous infusion or subcutaneous injections, even without intravitreal injection, thus reducing the systemic and local side effects documented with intravitreal antibodies such as ranibizumab [116,173,174]. Numerous types of molecules have been identified due to their major role over pathological angiogenesis, such as VEGF, nevertheless, considerable side effects have been clearly associated with anti-VEGF therapy, and thus other types of therapies should be proposed (Figure 1).

**Figure 2:**
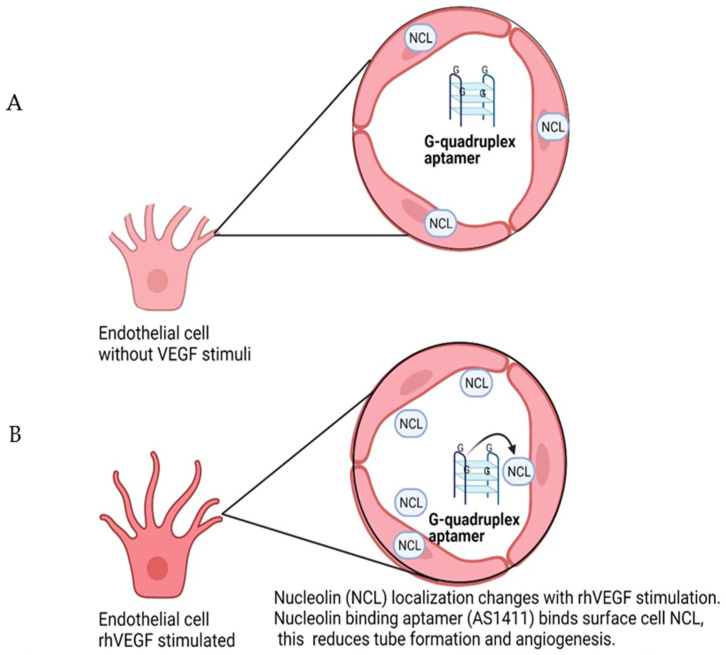
Nucleolin localization changes with rhVEGF in endothelial cells. (**A**) NCL is a protein mainly found in the nucleolus, but it can also be translocated to the cell surface by rhVEGF stimulation, therefore, nucleolin is not located on the cell surface of quiescent blood vessels, only in angiogenic vessels. (**B**) Huang. et al. demonstrated that rhVEGF, extracellular matrix, and the intracellular protein MyH9 are vital to mobilize NCL to endothelial cell surface membrane and therefore act as a proangiogenic factor [158]. Previous studies published by Darche et al. showed that retinal endothelial tip cells had NCL in surface membrane and demonstrated that intraperitoneal administration of a potential antagonist to NCL called N6L reduced retinal tuft in 50 % [159]. Created with BioRender.com.

A novel aptamer delivery system called aptamer-siRNA chimera delivers siRNA in a tumor-specific manner, reducing the cytotoxic effect over normal cells. Surface NCL serves as a tumor-specific drug delivery platform due to the overexpression of surface NCL in tumorigenic cells [175]. As shown in Figure 2, panel A illustrates an endothelial cell without VEGF stimulus, and therefore NCL localization is in the nucleus. Usually, NCL resides in the nucleus and the cytoplasm, but there is no NCL at the cell surface of endothelial cells. NCL localization changes mainly due to post-translational modifications such as N- and/or O-glycosylation. In Figure 2, panel B shows an endothelial cell stimulated with rhVEGF. VEGF stimulation induces NCL translocation into the endothelial cell surface due to post-translational modifications. Proliferative retinopathies are characterized by augmented levels of VEGF. The fact that surface NCL is constantly and abundantly expressed on the surface of tumor and endothelial cells could promote the shuttle of ligands between cell surface and the nucleus. Thereby, nucleolin-binding aptamers could be used as therapy or as a platform for delivery systems to reduce pathological angiogenesis in the eye, with the possibility of either intravenous infusion or subcutaneous injections in order to avoid intravitreal injections and ophthalmological side effects. 

## 8. Aptamers as Diagnostic, Therapeutic and Prognostic Agents

Aptamer-siRNA chimera has been used by conjugating nucleolin aptamer (aptNCL) to siRNAs against *SLUG* and *NRP1*. These genes promote malignant transformation, specifically, SLUG activates epithelial–mesenchymal transition (EMT) and lung cancer metastasis, while NRP1 functions as a co-receptor for VEGF and is involved in VEGF-downstream signaling. This therapy suppresses the expression and signaling of SLUG and NRP1 [176]. Another aptamer ARC5690, that is directed against P-selectin, reduces inflammation by preventing leucocyte rolling and adhesion to endothelial cells. Endothelial (E-selectin) and platelet (P-selectin) promote tumor cell adhesion to the vascular endothelium, and consequently, ASRC5690 is important to treat both metastases and inflammation [177]. 

E10030 is a DNA aptamer with 29 nucleotides that specifically recognizes PDGFB, and in this context, E10030 blocks ligand–receptor interactions. PDGFB stimulates cell proliferation, survival and migration, hence PDGFB upregulation has been associated to pathological angiogenesis, fibrotic conditions and atherosclerosis. This aptamer was chemically modified with the addition of a PEG molecule and 2′ ribose modifications (2′-F, 2′-OMe substitutions). In a randomized clinical trial, E10030 showed 62% visual improvements compared to ranibizumab [178,179,180]. PDGFB contributes to recruitment and maturation of pericytes. Thereby, blocking pericytes’ recruitment, survival and maturation with E10030 inhibits the development and maturation of newly formed vessels. Hence, a PDGFB antagonist offers a new treatment option for eye pathological angiogenesis, such as AMD [181]. 

ARC1905 is another interesting RNA aptamer, which contains 38 nucleotides and chemical modifications, such as PEG and 2′-F, 2′-OMe substitutions. ARC1905 specifically binds to the complement system molecule C5, inhibiting its function. It has been shown that complement triggers VEGF protein expression, and in consequence, angiogenesis [182]. Inhibition of C5 prevents the formation of the membrane attack complex (MAC), and therefore ameliorates the inflammatory microenvironment and diminishes retinal cell lysis. This aptamer has been used in clinical trials for AMD and idiopathic polypoidal choroidal vasculopathy [183]. 

The Tx-01 aptamer has been demonstrated to interact with heat shock protein-70 (HSP-70). Importantly, it has been proposed as a therapeutic and prognostic factor for serous ovarian cancer. HSP-70 is a chaperone protein that under stress conditions translocates to the nucleus [184]. This protein is highly expressed in cancer cells and is usually related with disease progression. Interestingly, HSP-70 overexpression has been associated with the aggressiveness of ovarian cancer [185]. It has been found on the plasma membrane in numerous types of tumors and thereby is used as a novel circulating tumor cell marker. In previous publications, authors showed that HSP-70 can interact with the Notch1 intracellular domain to activate Notch signaling. The Notch pathway is a highly conserved molecular cell pathway that regulates cell proliferation, stem cell maintenance, differentiation, survival and angiogenesis [186]. In this context, it has been demonstrated that therapeutic usage of aptamer Tx-01 significantly reduces HSP-70 translocation to the nucleus, and consequently, reduced cell migration and tumor invasion. The Tx-01 aptamer could be a potential candidate for ovarian cancer treatment with high levels of HSP-70 [187]. 

Other types of oligonucleotides have been used as therapeutic agents, such as antisense oligonucleotides (ASOs), including short-interfering RNA (siRNA). Antisense oligonucleotides are novel therapeutics designed to bind specific messenger RNA (mRNA) which exert their action by directly modulating target gene expression or function. In a recent phase I/II clinical trial, a specific ASO showed good results in treating Leber congenital amaurosis 10, Usher syndrome type 2 and (rhodopsin) RHO-associated autosomal-dominant retinitis pigmentosa [188]. Other studies using ASOs have confirmed that daily subcutaneous injection of antisense p21 oligodeoxynucleotide resulted in suppression of tumor growth and angiogenesis in highly metastatic mice breast cancer [189,190,191]. ASOs are short, single-stranded nucleic acids that offer some advantages over siRNAs, because they target cytoplasmic and nuclear located long non-coding (lnc) RNAs [192]. A second-generation antisense oligonucleotide, known as iCo-007, has been developed to target c-Raf kinase. Interestingly, c-Raf kinase is an important component of the mitogen-activated protein (MAP) pathway [193]. Multiple growth factors converge at the Ras/Raf/MAP signaling pathway, and therefore iCo-007 inhibits vascular permeability and angiogenesis [194].

## 9. Conclusions

The main goal in cancer and pathological angiogenesis medicine is to improve therapeutic efficacy by targeted delivery of antiangiogenic molecules. DNA aptamers are novel agents that are being used to improve therapeutic, diagnostic and prognostic outcomes, decreasing the undesirable side effects of conventional chemotherapy such as myelosuppression and cardiotoxicity [195,196]. AS1411 is an aptamer that binds specifically to NCL, discovered by Bates et al., by serendipity [197]. It has anti-proliferative effects, inhibits NF-κB-mediated pro-survival mechanisms, blocks DNA-replication and induces cell cycle arrest and apoptosis. Moreover, this aptamer has a lower immunological response compared to mAbs and has higher stability in serum samples [198]. Antiangiogenic intravitreal injections, even when performed under sterile conditions, present a risk of endophthalmitis, intraocular inflammation, vitreous hemorrhage, retinal detachment, intraocular pressure elevation, subconjunctival hemorrhage and chorioretinal atrophy, since VEGF is a pro-survival factor [199,200]. Furthermore, anti-VEGF ocular therapy can produce tachyphylaxis, and systemic side effects have been reported, including hypertension, stroke, thromboembolic events, myocardial infarction, gastrointestinal perforations and kidney disease [201]. Aptamers are novel molecules that can be delivered either by intravenous infusion or subcutaneous injections, reducing systemic and local side effects. Aptamers are non-toxic, non-immunogenic and have long-term storage at room temperature, thus, aptamer stability during shipping is warranted. Diverse possibilities of conjugating to other chemical reagents make aptamers novel molecules that could improve therapeutic outcomes [202]. One of the biggest challenges is to deliver efficiently diverse types of molecules such as gene-modifying agents and diverse chemotherapeutic agents to inhibit tumor and endothelial growth, and thus, molecular carriers are needed. Moreover, it has been demonstrated that AS1411 offers a positive impact in therapeutic nanomedicine due to the high ability of using it as a drug delivery system [203,204].

## Data Availability

Not applicable.
